# COVID-19 Effects on Public Finance and SDG Priorities in Developing Countries: Comparative Evidence from Bangladesh and Sri Lanka

**DOI:** 10.1057/s41287-022-00558-6

**Published:** 2022-07-27

**Authors:** Sisira R. N. Colombage, Suborna Barua, Madurika Nanayakkara, Udari N. Colombage

**Affiliations:** 1 Federation Business School, Federation University, Melbourne, Australia; 2 Department of International Business, University of Dhaka, Dhaka, Bangladesh; 3 Department of Commerce and Financial Management, University of Kelaniya, Kelaniya, Sri Lanka; 4 Monash University, Melbourne, Australia

**Keywords:** COVID-19, Sustainable development goals, Comparative study, Bangladesh, Sri Lanka

## Abstract

The COVID-19 pandemic, an unprecedented global health crisis, rapidly transferred into a global economic and social crisis. The pandemic has threatened the world’s commitment to achieve Sustainable Development Goals (SDGs) by 2030 as governments in developing countries have shifted their priorities from attaining SDGs, to providing urgent financial needs to save lives and prevent recession in hopes for a rapid economic recovery. The rerouting of public funding priorities has undermined the progress and achievement of SDGs. We employed a mixed-method and carried out a comparative study using pre- and post-public financial data of two developing countries in South Asia; Bangladesh and Sri Lanka. A threefold analysis was conducted to investigate the evolution of the COVID-19 pandemic in two countries, the impact of the pandemic on external and internal public finance and the effect of the pandemic in shifting the policy priorities from SDGs to economic survival. This study found that both countries are highly vulnerable to the COVID-19 pandemic and are suffering from the lack of financing from external sources through the private sector as well as an increasing foreign debt. There is mounting pressure on the fiscal balance in both countries.

**Supplementary Information:**

The online version contains supplementary material available at 10.1057/s41287-022-00558-6.

## Résumé

La pandémie de COVID-19, une crise sanitaire mondiale sans précédent, s'est rapidement transformée en une crise économique et sociale mondiale. La pandémie menace l'engagement mondial visant à atteindre les objectifs de développement durable (ODD) d'ici 2030, car les gouvernements des pays en développement ont changé leurs priorités, passant de la réalisation des ODD à la satisfaction des besoins financiers urgents pour sauver des vies et prévenir la récession, dans l'espoir d'une reprise économique rapide. Le changement de priorités et la réallocation des financements publics ont sapé les progrès et la réalisation des ODD. Nous avons utilisé une méthode mixte et réalisé une étude comparative à partir de données financières publiques pré et post dans deux pays en développement d'Asie du Sud, le Bangladesh et le Sri Lanka. Nous avons mené une analyse en trois volets pour étudier l'évolution de la pandémie de COVID-19 dans ces deux pays, l'impact de la pandémie sur les finances publiques externes et internes et l'effet de la pandémie sur le changement des priorités politiques, passant des ODD à la survie économique. Cette étude a révélé que les deux pays sont très vulnérables à la pandémie de COVID-19 et souffrent du manque de financements externes émanant du secteur privé ainsi que d'une dette extérieure croissante. La pression sur le solde budgétaire des deux pays est de plus en plus importante.

## Introduction

The unprecedented nature of COVID-19 pandemic has placed the global economy in crisis. The economic burden as a result of the pandemic continues to grow as it damage global supply chains, increase unemployment, decrease incomes, and raise poverty, while pushing economies toward recession. In the pre-COVID-19 era, achieving sustainable development was a primary focus of public policies in countries across the world. In early 2016, 17 sustainable development goals (SDGs) were officially launched by the United Nations (UN), and all countries were on track to achieve them by 2030. Since then, officials have prioritized the attainment of SDGs in both developed or developing countries. However, the emergence of the COVID-19 pandemic has brought a paradigm shift in the governmental policy priorities, particularly in developing countries. This is likely because the pandemic’s harshest impacts are faced by the poor and resource-starving developing economies. However, the growing literature on COVID-19’s multifaceted economic impacts offer ‘none’ to ‘little’ insight into how the pandemic places stress on the limited financial resources in developing economies. Thus, this has caused a shift in public funding priorities that undermine the progress and achievement of the SDGs.

The evolving literature show the potential implications for the SDGs based on the direct impacts of the pandemic on poverty, health, nutrition, etc. (The Lancet Public Health 2020; Barbier and Burgess 2020; Abidoye et al. 2021; Hughes et al. 2021). Other studies have investigated the impact of the pandemic redistributing finance generated for development purposes both from local and international sources across developing countries (e.g., Brown 2021; UN 2020; Blustein et al. 2020). However, these studies do not specifically and thoroughly analyze the potential public finance priority shifting that could hamper the progress and achievement of SDGs. An extensive evaluation is needed to comprehensively understand the changing landscape of public financing of SDGs and their priorities, particularly in developing countries.

Based on current evidence, this paper examines how the COVID-19 pandemic has placed pressure on the financial resources of developing countries, forcing governments to shift their priorities from the SDGs to the immediate concerns of saving lives and preventing recession, in hopes for a rapid economic recovery. The aggressive growth-first motive as followed by many developing countries is driving these countries away from the progress of SDGs; for example, through undermining human capital development, sustainable business practices and environmental protection, and improvement in health systems (Barua [Bibr CR11][Bibr CR12]; Barua and Aziz 2021). Generally, the lack of domestic financial resources is the major obstacle for developing countries in achieving the SDGs in time (Barua 2020a). The pandemic has introduced a significant shock to businesses and financial systems in developing countries, which has forced governments to spend billions in the form of stimulus packages, bailouts, direct cash transfers, employment supports, and others (Barua and Barua 2021). Such measures, continued for over a year, force many countries with limited resources to shy away from SDG-related priorities. SDG 1 no poverty, SDG 4 quality education and SDG 5 gender equality are particularly affected by the COVID-19 pandemic in Bangladesh and Sri Lanka. The economic downturn in both countries caused its citizens to become and remain poor. Furthermore, the transition from physical to digital education increased the inequality in education between urban and rural areas as majority of people in rural areas were poverty-stricken and experienced difficulties with accessing technology. Only 45% of students in government owned schools accessed internet-based education in Sri Lanka during the lockdown period. Comparatively in Bangladesh, whereas only 11% had access to internet facilities (Gamage and Zaber 2021). Similarly, the shift to working from home led to more pressure on working women to balancing office work, children’s education, and household maintenance from home.

This paper critically evaluates how the shifts in funding priorities are likely to threaten the progress and achievement of SDGs in developing countries—specifically Bangladesh and Sri Lanka. There are several reasons to why these two countries provide significantly divergent yet important lessons from the rerouting of public finance away from SDG priorities. Although Bangladesh and Sri Lanka have close economic and geographical ties, they show significantly divergent patterns of socioeconomic development and challenges (Rahman et al. 2019). On economic terms, Bangladesh has shown significant resilience over the last decade and has emerged as one of the leading economies. Comparatively, the Sri Lankan economy significantly lag behind. Despite the economic divergence, both countries have similarities in the healthcare systems such as GDP expenditure for health (2.3% in Bangladesh and 3.7% in Sri Lanka) and life expectancy (73 years in Bangladesh and 77 years in Sri Lanka) (The World Bank 2021). Given the close similarities in health quality and health systems investments, the significantly divergent pattern of economic progress makes it interesting to understand whether the differences in economic strengths explain how each country navigated through the pandemic.

We adopted a threefold approach in analyzing the public finance priority shift in Bangladesh and Sri Lanka. Our findings suggest that both countries are under high pressure to maintain the fiscal balance as a result of low financing flows from external sources. In turn, both countries are struggling to reach SDGs due to changing priorities during the COVID-19 pandemic.

This paper makes several contributions to the evolving but limited literature on COVID-19 and SDG financing in developing countries. First, our analysis provides insight on the extent and degree the pandemic has impacted on the patterns of internal and external financing from different sources. Second, our paper provides detailed evidence on the policy responses implemented by each country to mitigate the detrimental health outcomes of COVID-19 leading to stress on financial resources. Third, the paper distinctively identifies the shift in the SDG-related policy priorities that have already taken place and are likely to happen, as countries divert more funding toward saving lives and the economy. All considered, this paper provide novel contributions to understanding how the COVID-19 pandemic pushes developing countries’ focus away from the SDGs, which is likely to delay the SDG’s achievement by 2030.

The remainder of the paper is organized as follows: “Evolution of Covid-19 in Bangladesh and Sri Lanka” section describes the evolution of the pandemic in both countries; “Methodology” section outlines the methodology followed by a theoretical framework for the study in “How COVID-19 Could Affect SDG Priorities: A Theoretical Framework” section; “Analysis of Key Public Finance Sources for Bangladesh and Sri Lanka” and “Are Financing Priorities for SDGs Shifting?” sections analyze the key sources of public finance for the two countries investigated in the study and demonstrate the impact of the pandemic on SDG priorities and finally, “Conclusion” section offers the conclusion and future research priorities.

## Evolution of Covid-19 in Bangladesh and Sri Lanka

This section briefs the evolution of COVID-19 in Bangladesh and Sri Lanka, both of which were highly affected by the pandemic. Bangladesh reported the second highest number of cases in South Asia in 2020. Conversely, Sri Lanka was ranked the 9th best country in the world which controlled COVID-19 in 2020 by the Global Responses to Infectious Diseases (GRID) Index developed by Institute of Certified Management Accountants of Australia (Daily Mirror 2020). However, both countries were identified as highly vulnerable countries to COVID-19 in 2021.

### Evolution of COVID-19 in Bangladesh

**Fig. 1:**
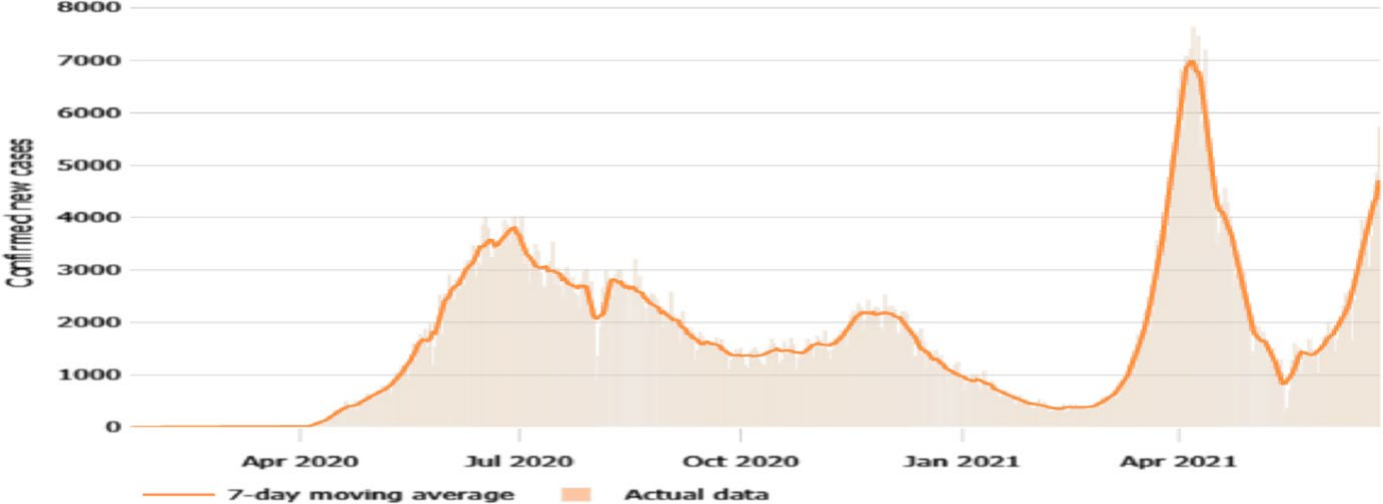
Bangladesh daily new confirmed cases, 7-day moving average. *Source*: John Hopkins University Coronavirus Resource Center, accessed June 25, 2021

Bangladesh was one of the most adversely affected countries in South Asia by the pandemic. The first three COVID-19 infections were confirmed on March 8, 2020 by the country’s Institute of Epidemiology, Disease Control and Research (IEDCR). To combat the COVID-19 outbreak, the government closed all educational institutions (schools, colleges, and universities) on March 16, 2020. Bangladesh announced its first COVID-19 death on March18, 2020. On 23 March, Bangladesh government announced general holidays which came into effect on 26 March and was previously scheduled to remain in place until April 4th. Bangladesh had recorded 51 confirmed cases and five deaths by the end of March. On April 5, 2020, the Government of Bangladesh launched the National Preparedness and Response Plan for COVID-19, which was created based on the country's prior experience and WHO recommendations. Since 16 March 2020, Bangladesh has seen nationwide lockdowns for an extended period to date, although the restrictions have become relatively relaxed over time. While all transportation through all modes—water, land, and air—were strictly closed initially, only air transport remains closed to date. According to the IEDCR, as of June 1, 2020, there are 49,534 confirmed COVID-19 cases in Bangladesh, including 672 fatalities; leading to a Case Fatality Rate (CFR) of 1.36 percent. The total number of infected cases reached 848,000 in June 2021, of which 780,000 recovered and 13,466 died. Bangladesh began administering COVID-19 vaccinations on January 27, 2021, with widespread immunization beginning on February 7, 2021. From January through April 2021, the only COVID-19 vaccine approved for emergency use was the Oxford–AstraZeneca vaccine. According to John Hopkins University data, as of June 25, 2021, the country has reported 866,877 cases and 13,787 deaths. Figure 1 presents the trend of daily new COVID-19 confirmed cases in the country.

According to Islam et al. (2020), Bangladesh, a highly populated developing (lower-middle income) country with an overall population of 161.3 million, is facing a calamity as a result of the pandemic. Bangladesh is more prone to the transmission of the virus due to its high population density when compared to nations with lower population density. Bangladesh has made a number of efforts to combat the effects of the pandemic. Lifestyle adjustments, the use of face masks, movement restrictions, social distancing, and changes in hygiene habits are among these policies. According to Islam (2020), the shutdown of public services and daily activities triggered an economic crisis in addition to the health catastrophe. One-fifth of the country's population lives in poverty, and a sizable part of the workforce is reliant on temporary labor. The closure posed a dilemma between protecting lives and preserving livelihoods. The country's largest manufacturing business of readymade garments (RMG) employs around 4 million individuals, many of them with unstable work arrangements (ILO 2016). When COVID-19 expanded to developed nations that are key importers of RMG sector products, customers began to cancel their orders, threatening the livelihoods of employees in the business. According to the Bangladesh Labour Force Survey (LFS) 2016–17, out of the total 60.83 million employed labor in the country, 85.1 percent work in the informal sector (Bangladesh Bureau of Statistics 2020). Their working circumstances are no better than those of casual laborers. Furthermore, amid the significant number of self-employed working individuals, around 5.19 million self-employed persons in the urban informal sector live in hazardous circumstances.

**Fig. 2:**
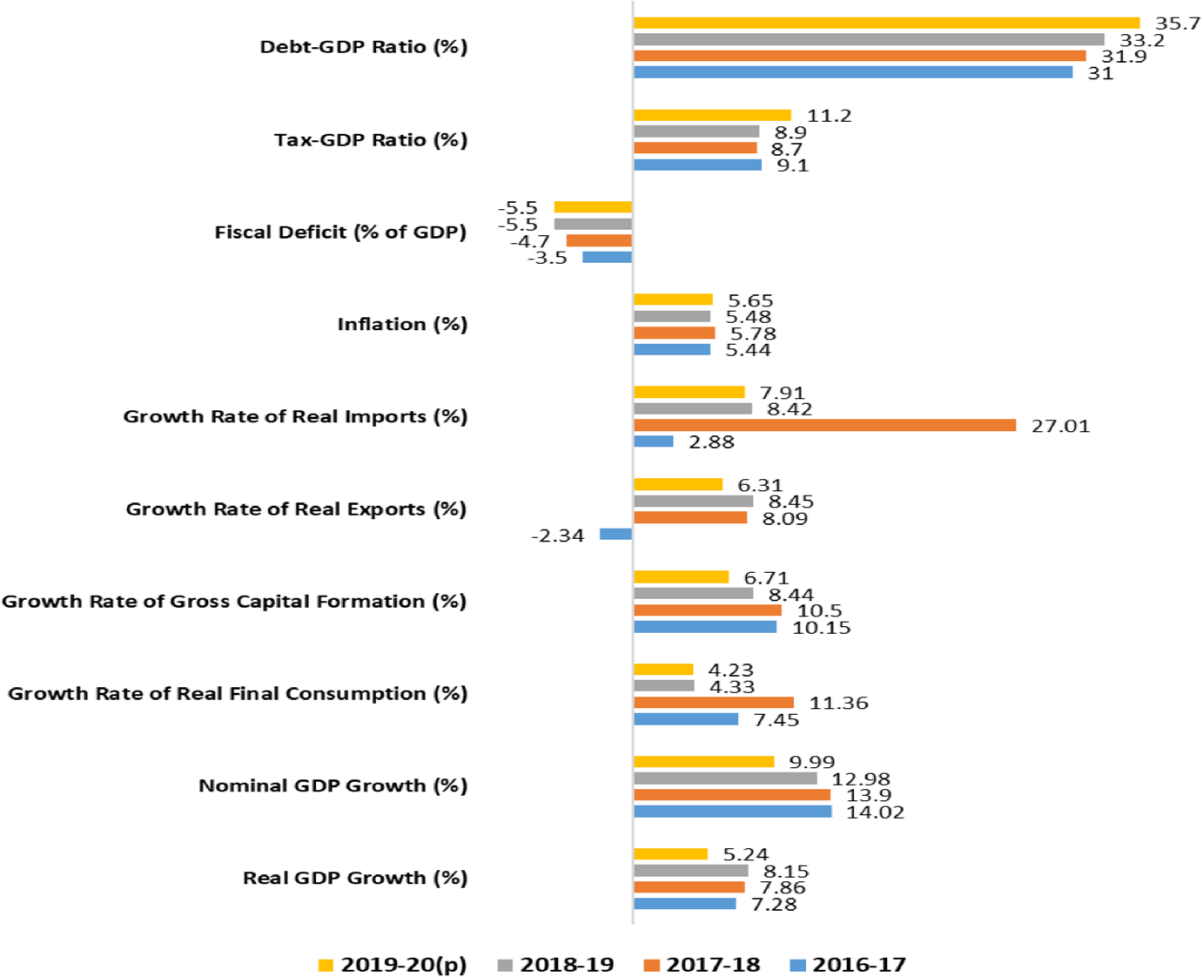
Bangladesh pre- and post-COVID key economic indicators. *Source*: Developed based on Siddiquee and Faruk (2020)

Figure 2 shows that economic growth is much lower in FY2019-20 than in the preceding years due to COVID-19 impacts. Final consumption growth declined significantly, mainly due to the lower growth of general government spending in FY2019-20. Investment growth was at a modest 6.71% in real terms in the year. International trade declined significantly as both exports and imports simultaneously dropped, reflecting the decline in international and domestic demand for goods. However, Bangladesh's economy shows resilience as fiscal deficit, debt–GDP ratio, inflation, and foreign exchange reserves balance indicates macroeconomic soundness to date.

### Evolution of COVID-19 in Sri Lanka

Similarly, Sri Lanka was identified as a country vulnerable to the effects of COVID-19 in 2021. The smaller number of testing sites in the country in response to the rapid increase in the number of cases and deaths, is highlighted and criticized in many national and international forums.

As per the Health Promotion Bureau of Sri Lanka, the first confirmed case of COVID-19 was reported on January 27, 2020 and another five suspected cases were reported on the following day. The first infected person was a tourist from China who fully recovered on February 19, 2020. The five other suspected cases were not diagnosed with COVID-19. Since the first confirmed case, 2798 confirmed cases including 80 deaths were reported in Sri Lanka.

On March 20, 2020, the Sri Lankan government imposed an island wide lockdown to control the spread of the virus. As a result of the lockdown, Sri Lanka was able to successfully control the situation and was recorded as the 9th best country to respond to the pandemic in the world. Accordingly, the lockdown was lifted on May 11, 2020. The number of cases then unexpectedly increased in July 2020. However, the situation was controlled within a shorter period through similar measures. While health outcomes improved by the end of July 2020, the Sri Lankan economy had its worst hit due to border closures which restricted access for tourists.

The second wave of COVID-19 in Sri Lanka started in October 2020, and continued till January 2021. The wave began with the majority of workers at an export garment factory testing positive for COVID-19. Despite this, the Sri Lankan government did not place an island wide lockdown, as the Sri Lankan economy was yet to recover from the first lockdown. The number of confirmed cases and resultant deaths continued to escalate. A quarantine curfew was imposed, mainly for the Western and North Western province. This was lifted in March 2021, allowing people to travel between provinces. Additionally, the vaccination program started in the country after obtaining the approval for Oxford–AstraZeneca Vaccine in January 2021.

**Fig. 3:**
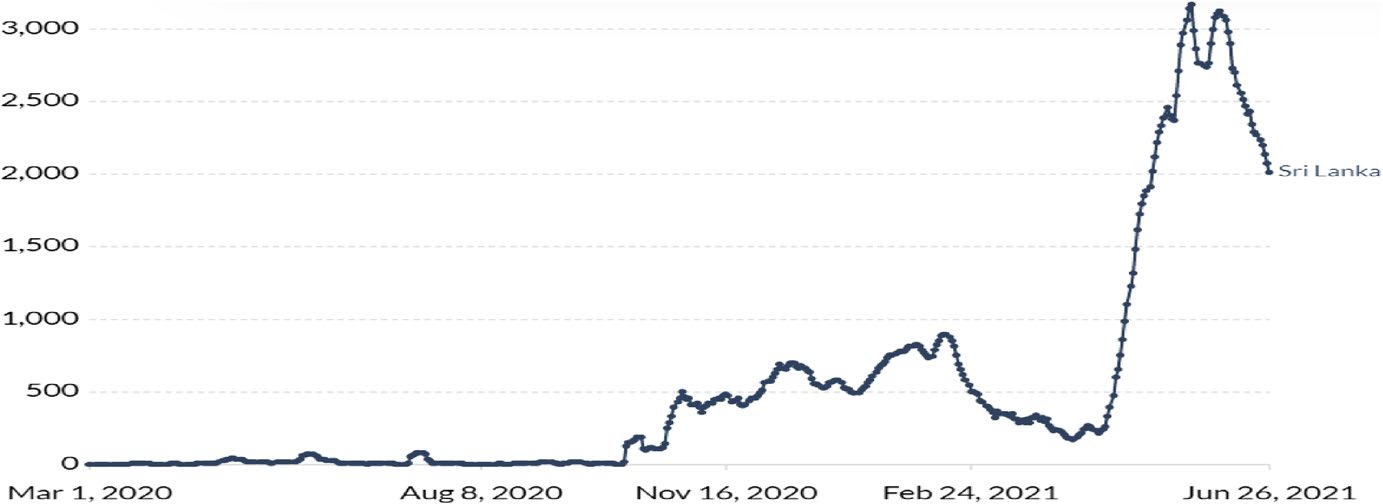
Sri Lanka daily new confirmed cases, 7-day moving average. *Source*: John Hopkins University Coronavirus Resource Center, accessed June 27, 2021

After the New Year festival in April 2021, Sri Lanka faced the third wave of COVID-19 and it is reported as the worst wave in the country. As of June 2021, there are 251,751 total confirmed cases, including 218,998 number of recovered cases and 2905 deaths. Fatality rate is reported as 1.15% with a recovery rate of 83.83%. Reports also suggest that 3.98% of the total population is vaccinated as of June 2021. Figure 3 depicts the daily new confirmed cases, (7-day moving average) in Sri Lanka.

Undoubtedly, the COVID-19 pandemic has taken a toll on the Sri Lankan economy. Lockdowns led to a substantial downturn in the country’s key economic sectors, such as agriculture, tourism and hotel services. Purchasing Manager’s Index (PMI) of Manufacturing sector is reported as 42.1 in May 2021 showing a decrease of 2.2 index points compared to the previous month. Furthermore, the PMI of the service sector in May 2021 was recorded 9.4 index points less than the index point value in April 2021 (CBSL 2021). Moreover, Sri Lanka’s GDP, decreased by 3.6 percent in 2020, while the Gross National Income recorded a decrease of 5% compared to 2019. Further, both industrial exports and agricultural exports declined significantly in 2020 compared to the previous year. The ratio of central government debt service payments to government revenue degraded to 141.9 per cent in 2020 from 107.0 per cent in 2019, mainly due to the drastic decline of revenue in 2020 as a result of the downturn in the total economy and the tax concessions granted in late 2019. On top of that, the significant decline in the private remittances of migrant workers also destructively impacted the economy of Sri Lanka (CBSL 2021).

## Methodology

Our analysis follows a threefold approach: first, we examined the evolution of the pandemic in the two countries since the first identified case; second, we evaluated how the evolution of the pandemic over time has impacted the countries’ patterns of external and internal financing from four key sources: ODAs, tax revenues, remittances, and FDIs; and third, we reviewed the governments’ policy responses and identified how policy priorities shifted from SDGs to economic survival, which in turn undermines the progress and achievement of the SDGs. In ODAs, we examined the flows of foreign loans and grants, debt repayments and outstanding debt balances separately. As an additional measure to capture foreign earnings, we evaluated export receipts. In tax revenues, we examined the flows of income tax, value added tax (VAT), excise duties, import duties, airport levy, and other taxes and surcharges that make up the total tax revenues for the governments in the two countries. We adopted a mixed-method approach combining content analysis, descriptive analysis, and the application of statistical tools on data from relevant sources, including the World Bank, the International Monetary Fund, and the UN system organizations and government databases. Furthermore, to test whether flows of domestic and external financing is significantly affected due to the COVID-19 pandemic, we implemented the two-sample mean difference test. All comparisons were carried between two periods—pre- and post-COVID-19 (defined as the on-going COVID period), e.g., before January 2020 and since January 2020 to date.

## How COVID-19 Could Affect SDG Priorities: A Theoretical Framework

The COVID-19 pandemic’s toll will stretch out across both micro and macro levels, resulting in a significant pressure on public financial resources. Although the ultimate outcome of continued adverse economic impacts could be a long-lasting recession, the effect mechanism, (i.e., how a pandemic affects economic activities and agents) is likely to be more or less different compared to other known events resulting in a similar outcome, such as the 2008–09 global financial crisis. One peculiarity of the current case is that the end to the pandemic remains uncertain, and thus the direst consequences are likely to arise in the long-run if the pandemic continues to devastate these countries.

The SDGs are a combination of 17 intertwined global goals designed to ensure a more sustainable future of the world included in the UN Resolution called the 2030 Agenda (UN 2017). The UN General Assembly officially adopted the SDGs in 2015, where all countries under the UN aimed to achieve the goals successfully by 2030. The 17 goals can also be considered a post-development advancement of the Millennium Development Goals that ended in 2015. The 17 SDGs are: (1) No Poverty, (2) Zero Hunger, (3) Good Health and Well-being, (4) Quality Education, (5) Gender Equality, (6) Clean Water and Sanitation, (7) Affordable and Clean Energy, (8) Decent Work and Economic Growth, (9) Industry, Innovation and Infrastructure, (10) Reducing Inequality, (11) Sustainable Cities and Communities, (12) Responsible Consumption and Production, (13) Climate Action, (14) Life Below Water, (15) Life On Land, (16) Peace, Justice, and Strong Institutions, (17) Partnerships for the Goals. Each of the SDGs have its own strategic goals which are targeted to eventually lead to the achievement of SDGs.

We developed a theoretical mapping of the COVID-19 implications for public finances, extending the theoretical framework of Barua (2021c). Based on Barua (2021c), Fig. 4 shows a general theoretical mapping of the likely economic impacts of the COVID-19 pandemic for public financial resources and SDG priorities; showing what ‘could be’ the impact span and progress line. It is useful to consider the mapping in the macroeconomic context of a developing economy. The figure assumes different waves of impacts over time, where many of the impacts could be visible in the short run while others in the long-run. Furthermore, several impacts could happen concurrently, while others sequentially. To begin with the first wave, the immediate and direct impact of the COVID-19 pandemic is the temporary shutdown of factories and businesses in an affected country (as it is the case in China) and thereby, resulting in a sharp and immediate decline of production in the economy (Barua S. 2020b; Barua and Nath 2021). The shock then could be amplified by simultaneous supply chain disruptions of necessary production inputs and immediate drop in demand. The demand for goods and services declines as consumers follow ‘saving for emergency’, ‘wait and see’, and ‘hoarding’ during the crisis (Baldwin and Tomiura 2020). Also, as the pandemic spreads across the world, foreign demand for an economy’s goods may slump substantially, which in turn will depress production (creating a situation termed as supply–demand doom loop described by Fornaro and Wolf, (2020)). However, there is an important aspect of the demand response. To worsen the situation, lower production and supply (driven by demand drops, manufacturing hits, and supply chain disruptions) sends back some effects to the global supply chain, particularly when the rest of the world significantly relies on the affected economy (such as China) for production inputs. In addition, when domestic and international transports and logistics channels are suspended by the country in an effort to stop the pandemic’s spread, it could further disrupt the supply chain. And if the affected economy is a world’s major manufacturing-supply hub like China, a continued disruption could eventually breakdown or collapse the global supply chain. Furthermore, both production and supply chain would be substantially interrupted due to no to fewer human movement, both domestically and internationally, as restrictions and border closures are imposed. Globally, restrictions of people movement and lockdown are considered to be the most effective way in preventing the transmission of COVID-19. It is worth noting that all these effects in the first wave could be interrelated to some extent and occur concurrently in the affected economy.

**Fig. 4:**
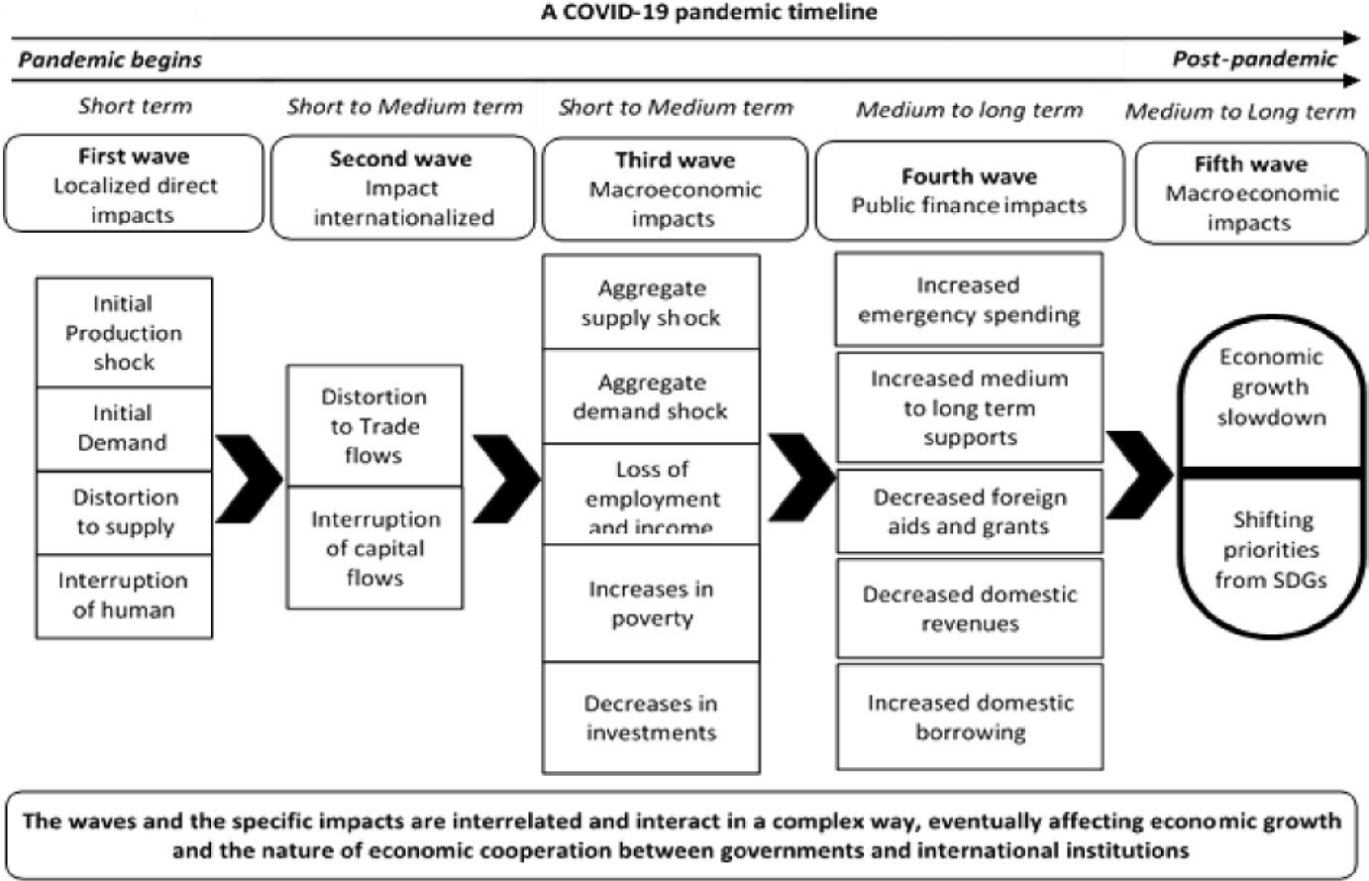
A pandemic timeline to visualize the COVID-19 implications for SDG priorities

A concurrent shock to the country’s production, demand, supply chain, and human flows is likely to result in a significant reduction in international trade flows of goods and services (the second wave); for example, a reduced supply, transport routes closures, lower demand for imported goods, and lesser movement of people from one country to the other—all could significantly reduce exports and imports of both goods (e.g., manufactured products such as automobiles) and services (e.g., tourism, traveling) for the economy. Furthermore, fewer people movement, economic uncertainty, and interrupted transports and logistics—coupled with higher costs of available options due to interruptions—could in combination force international investors to hold back on their on-going investment activities and plans in the pandemic-affected country (Baldwin and di Mauro 2020). In general, this could hit resource-scarce developing economies the hardest as they rely heavily on trade and foreign direct investments inflows for economic growth and development.

The effect of the pandemic on specific macroeconomic indexes could be delayed and take some time to reflect. A continued distortion in the production, supply chain, demand, flows of human, trade, and investment combined for a prolonged period will begin damaging the macroeconomic indicators of the affected economy (Baldwin and di Mauro 2020). The first of the hits should be a sizable reduction in aggregate supply and demand in the economy. The production shock discussed earlier explains the reduction in aggregate supply. With respect to aggregate demand, while demand for essential goods may increase (e.g., people may hoard food and medicine to face the uncertainty, more medicines are needed as more people become sick due to the pandemic), that of non-essential goods will reduce sharply (as people tend to save for the emergency, suspend non-essential consumption, and spend only for the need of the hour) (Barua 2021c). This should lead to a net fall in the aggregate demand of the economy. The production and demand falls will eventually instigate a fall in fresh investment and increases in divestments. Firms, under these circumstances, would be forced to lay-off a large number of workers in an effort to survive and remain financially feasible, which could significantly increase unemployment in the economy (Blustein, 2020). This is likely as firms will face a slump in business volume and revenues and increases in costs, which may lead to many firms not being able to service their wage payments and debts. The overall outcome of these effects is an increase in unemployment rate and decrease in incomes of the people in the affected developing economy.

Reduced production, incomes, consumption, investments, and international trade would result in a significant decline in domestic public revenues in the form of taxes and surcharges. As all countries across the world face similar consequences and shift resources to focus on fighting the pandemic and economic recovery, cross-border financial assistance, in the forms of ODAs such as grants, loans, and aids, from developed countries to developing countries are likely to decline significantly (Brown 2021). On the other hand, faced with the expanding COVID-19 health system shocks and efforts to save both lives and the economy, public spending of the developing country increases in the forms of direct and indirect supports (cash and in kind) to the poor and the labor market, and immediate stimulus and bailout packages for businesses and industries. Much of the supports are likely to extend over a medium to longer term period to facilitate the domestic industries and businesses to survive the pandemic and contribute to economic recovery (UN, 2021). Decreased public revenues, foreign assistance, and increased immediate and medium-term spending—all combined are likely to quickly deplete existing public financial resources (OECD 2020; UN 2020). It will force the developing country government to borrow more from the local market to finance the expanding fiscal gap (OECD 2020; UN, 2021). While the government struggles to keep spending now as well as for the future, and keep borrowing to finance the fiscal gap, the government’s spending priority is likely to change from what they were in the pre-pandemic era. Public spending priorities are likely to be diverted to save lives and continue the economic recovery mechanisms from those that are not essential in fighting the pandemic’s effects. Many of the SDGs (e.g., Life under water or Sustainable Cities) that generally interest the government may now be deprioritized, as they are not likely to be considered seriously essential for fighting the pandemic (OECD 2020; UN, 2021). However, some public spending (e.g., direct or indirect support to the poor) during the pandemic might have an implication for some of the SDGs (e.g., No Poverty).

## Analysis of Key Public Finance Sources for Bangladesh and Sri Lanka

For developing countries like Bangladesh and Sri Lanka, financing SDGs is largely dependent on external sources, such as grants, loans, and aids. For example, Bangladesh requires about USD 964.72 billion to achieve the SDGs, in which the government and stakeholders aim to reach at an average sectoral contribution of 34%, 41%, and 15% from public, private and external sources, respectively, from 2020 to 2030 (GoB, 2020). However, the current situation remains far short of the aim, which can be understood by looking at its heavy fiscal reliance on external sources. Presently, about 48% of the government’s ADP budget (USD 24.2 billion) is being financed by external sources (project aid 35% and foreign borrowing 13%), while the rest is financed by revenue surplus (27%) and domestic borrowing (25%) (CPD, 2020). One of the dangers of relying on external sources is that it can dwindle given any changes in national and international economic scenarios. For example, the recent COVID-19 crisis has contracted the loan and grants funds for Bangladesh up to 46% compared to the same period in the previous fiscal year (Holy and Rozario, 2020).

**Fig. 5:**
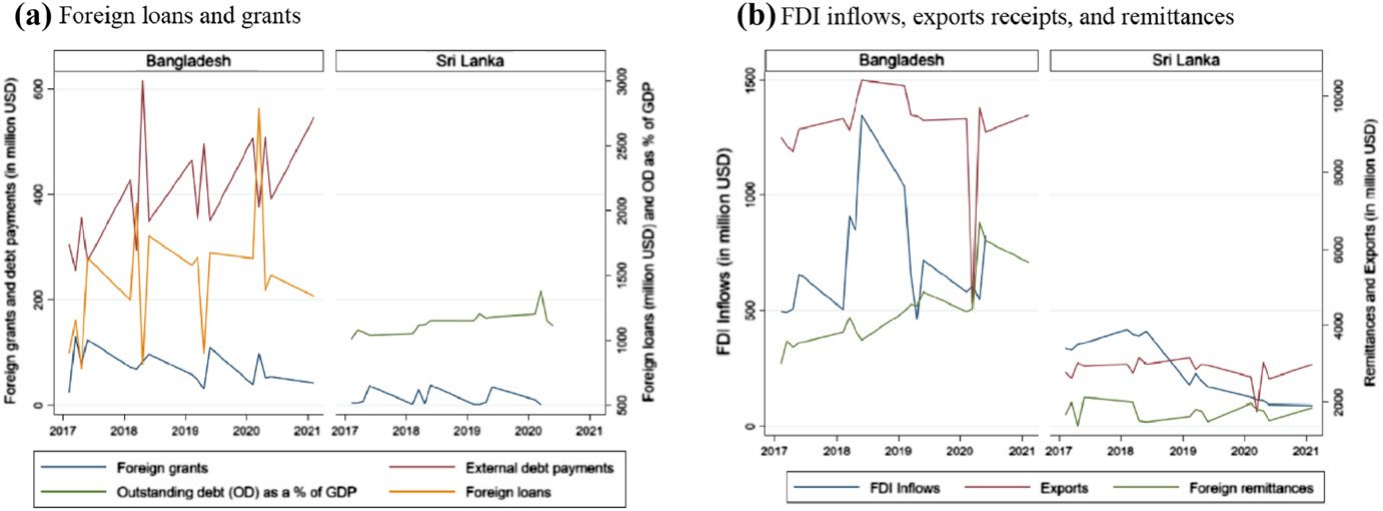
Quarterly trends of inflows from external sources, 2017–2021 (Q1). (a) Foreign loans and grants(b) FDI inflows, exports receipts, and remittances. *Source*: Authors’ developed based on published government data

Figure 5 presents how the pandemic affect the trends of quarterly external financing from various sources in both countries. For foreign loans, external debt payments, and outstanding debt as % of GDP – data were not available for both the countries. Figs. (5a) and (5b) show that both foreign loans and grants have dried up since Q1 2020 to Q1 2021 compared to the levels in the earlier years. In addition, foreign debt repayments showed an increasing trend for Bangladesh. On the other hand, FDI inflows and exports receipts appear to decline in the middle of 2020, with signs of recovery in the later quarters. While remittances continue to improve for Bangladesh, it consistently declines for Sri Lanka. The graphs together suggest some recovery in the private sector foreign flows after the immediate shock of COVID-19, while financing that serve as the main external sources of public finance (e.g., foreign loans and grants) tend to decline significantly. However, the private sector external financing remains significantly vulnerable depending on the spread of COVID-19 cases, vaccination rates, and continuation of economic supports of the governments. As such, if the countries cannot contain the spread of the virus, improve herd immunity through mass-vaccination, and keep supporting businesses with economic policies, private sector flows may see a significant decline in upcoming periods.

**Fig. 6:**
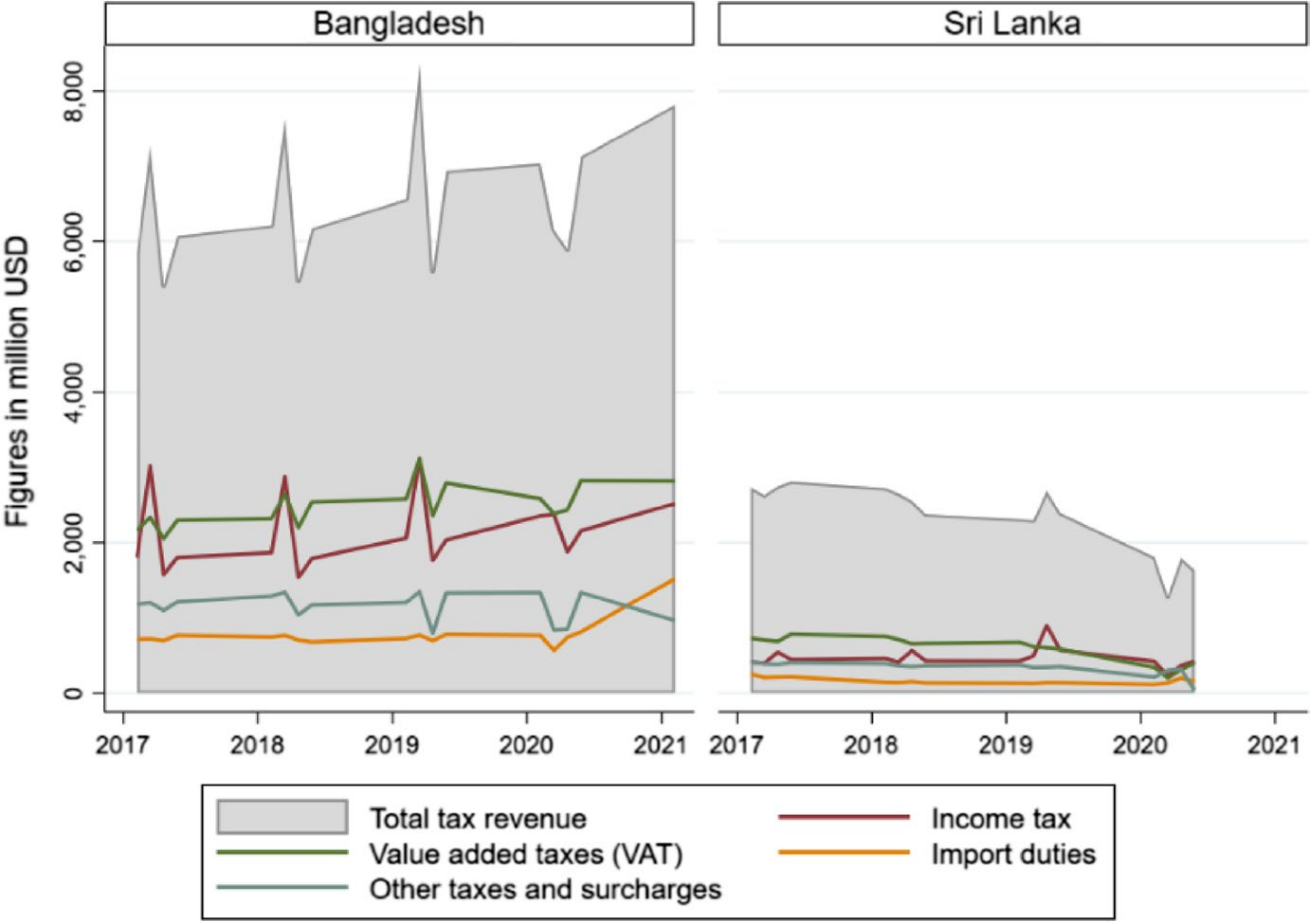
Quarterly trends of tax revenues from domestic sources, 2017–2021 (Q1). *Source*: Authors’ developed based on published government data

Figure 6 shows the governments’ tax revenues from domestic sources as the main source of public finances. Total tax revenues consistently decline during the COVID period for Sri Lanka, while it appears to increase for Bangladesh. In line with this patterns, income taxes and value added taxes—the two main sources of total tax revenues—tend to consistently decline during the COVID period in Sri Lanka. They tend to show slight improvement for Bangladesh in the later part of the COVID period. Overall, with a consistently declining patterns of domestic tax revenues, Sri Lanka’s fiscal struggle continues to intensify.

It is difficult to ascertain the effects on public financing from both external and domestic sources due to the COVID-19 pandemic as shown in Figs. 5 and 6. In order to examine the effects of COVID-19, we applied two-sample *t* test of mean difference on the quarterly average inflows by grouping them into two time frames—Q1 2017 to Q4 2019 as pre-COVID and Q1 2020 to Q1 2021 as post-COVID. Tables 1 and 2 show the mean difference test results for all sources of public finance for which data were available. Table 1 shows that financing from almost all domestic sources increased during the COVID-19 period as the differences between pre- and post-COVID-19 quarterly average values are positive. However, the differences found were not statistically significant. In other words, although financing from the domestic sources tend to increase for Bangladesh on a quarterly basis, it does not show a significant improvement. On the other hand, for Sri Lanka, the situation is opposite. Quarterly financing from all domestic sources have declined on average, as the mean differences for all sources are negative. Except for import duties and airport levy, tax revenues (e.g., total tax revenues, including income tax, VAT, excise duties, other taxes and surcharges) have dried up significantly during the COVID period for Sri Lanka. The largest decline has happened for VAT, followed by excise duties. Table 1 overall indicates a significant fiscal pressure particularly for Sri Lanka, while for Bangladesh the improvements remain non-significant.

**Table 1:** COVID-19 effects on public financing from domestic sources

		Sri Lanka			Bangladesh		
	A	B	C	B-C	E	F	E–F
	Particulars	Since 2020	2017–2019	Difference	Since 2020	2017–2019	Difference
Income Tax	Obs	5	12		5	12	
	Mean	359.8	503.3	− 143.5**	2253.6	2103.3	**150.2**
	Std. Err	45.6	39.7	74.5	111.4	163.2	266.6
	Std. Dev	91.1	137.5		249.1	565.4	
	[95% Conf	214.8	416.0	− 303.2	1944.3	1744.1	− 418.1
	Interval]	504.8	590.7	16.2	2562.8	2462.6	718.6
Value added taxes (VAT)	Obs	4	12		5	12	
	Mean	313.7	680.5	− 366.8***	2607.9	2448.9	**159.0**
	Std. Err	42.4	17.4	38.3	93.2	87.0	148.8
	Std. Dev	84.8	60.3		208.5	301.3	
	[95% Conf	178.7	642.2	− 448.9	2349.0	2257.4	− 158.2
	Interval]	448.7	718.8	− 284.6	2866.8	2640.3	476.3
Import Duty	Obs	4	12		5	12	
	Mean	153.4	168.8	**− 15.5**	881.8	730.8	**151.0**
	Std. Err	18.1	12.2	23.7	165.2	9.8	102.7
	Std. Dev	36.2	42.2		369.3	34.0	
	[95% Conf	95.7	142.0	− 66.2	423.2	709.2	− 67.9
	Interval]	211.1	195.7	35.3	1340.3	752.4	369.9
Excise Duty	Obs	4	12				
	Mean	431.6	686.1	− 254.6***			
	Std. Err	54.6	32.8	65.0			
	Std. Dev	109.3	113.5				
	[95% Conf	257.7	614.0	− 394.1			
	Interval]	605.4	758.3	− 115.1			
Airport Levy	Obs	4	12				
	Mean	154.9	165.1	**− 10.2**			
	Std. Err	11.3	2.9	8.0			
	Std. Dev	22.6	10.1				
	[95% Conf Interval]	119.0	158.7	− 27.2			
		190.9	171.5	6.9			
Others Taxes	Obs	4	12		5	12	
	Mean	218.4	373.4	− 154.0***	1064.3	1183.5	**− 119.2**
	Std. Err	64.1	6.9	36.4	113.4	45.2	99.7
	Std. Dev	128.1	24.1		253.5	156.5	
	[95% Conf	14.6	358.1	− 233.0	749.5	1084.1	− 331.7
	Interval]	422.3	388.7	− 76.9	1379.1	1282.9	93.3
Total Tax Revenues	Obs	4	12		5	12	
	Mean	1631.9	2577.3	− 945.4***	6807.5	6466.5	**341.1**
	Std. Err	121.9	53.9	115.6	349.0	265.2	470.5
	Std. Dev	243.7	186.6		780.4	918.7	
	[95% Conf	1244.0	2458.7	− 1193.4	5838.6	5882.8	− 661.8
	Interval]	2019.7	2695.9	− 697.5	7776.5	7050.2	1343.9

*Source*: authors’ developed based on published government data;

Significance levels: *** = 1%, ** = 5%, and * = 10%

**Table 2:** COVID-19 effects on public financing from foreign sources

		Sri Lanka			Bangladesh		
	A	B	C	B-C	E	F	E–F
	Particulars	Since 2020	2017–2019	Difference	Since 2020	2017–2019	Difference
FDI Inflows	Obs	5	12		4	12	
	Mean	104.6	312.0	− 207.4***	640.9	719.9	− 79.0***
	Std. Err	6.2	27.4	43.4	63.5	78.6	143.5
	Std. Dev	13.9	94.9		126.9	272.4	
	[95% Conf	87.3	251.7	− 300.0	438.9	546.8	− 386.8
	Interval]	121.8	372.3	− 114.8	842.9	893.0	228.8
Remittance	Obs	5.0	12.0		5	12	
	Mean	1794.2	1741.3	**52.9**	5479.1	3954.4	1524.7***
	Std. Err	74.7	70.8	120.9	472.1	160.5	385.3
	Std. Dev	167.1	245.4		1055.6	556.0	
	[95% Conf	1586.7	1585.4	− 204.8	4168.4	3601.2	703.5
	Interval]	2001.7	1897.2	310.6	6789.7	4307.7	2345.8
Foreign Grants	Obs	5	12		2	12	
	Mean	57.0	76.6	− 19.6	5.63	14.08	− 8.46
	Std. Err	10.6	9.8	16.8	4.76	4.43	11.33
	Std. Dev	23.8	34.0		6.73	15.36	
	[95% Conf	27.5	55.0	− 55.5	− 54.87	4.32	− 33.13
	Interval]	86.5	98.2	16.3	66.12	23.84	16.22
Foreign Loans	Obs				5	12	
	Mean				1730.0	1354.4	375.6*
	Std. Err				270.2	125.9	259.0
	Std. Dev				604.2	436.0	
	[95% Conf				979.8	1077.3	− 176.4
	Interval]				2480.2	1631.4	927.7
Outstanding Debt as % of GDP	Obs	4	12				
	Mean	174.9	150.4	24.5**			
	Std. Err	14.8	4.5	11.2			
	Std. Dev	29.5	15.5				
	[95% Conf Interval]	127.9	140.5	0.5			
		221.8	160.2	48.5			
External Debt Repayment	Obs				5.0	12.0	
	Mean				466.0	378.4	87.6*
	Std. Err				34.5	30.3	52.3
	Std. Dev				77.2	104.9	
	[95% Conf				370.1	311.8	− 23.9
	Interval]				561.9	445.0	199.1
Total ODAs	Obs				5	12	
	Mean				1787.0	1431.0	**356.0**
	Std. Err				280.1	129.0	266.7
	Std. Dev				626.4	446.7	
	[95% Conf				1009.2	1147.2	− 212.3
	Interval]				2564.8	1714.8	924.4
Exports	Obs	5	12		5	12	
	Mean	2605.8	2932.5	− 326.6**	8451.2	9383.5	− 932.3*
	Std. Err	227.6	46.7	158.1	973.7	164.0	652.1
	Std. Dev	508.8	161.7		2177.4	568.2	
	[95% Conf	1974.0	2829.7	− 663.6	5747.6	9022.5	− 2322.3
	Interval]	3237.6	3035.2	10.4	11,154.8	9744.5	457.7

*Source*: authors’ developed based on published government data

Significance levels: *** = 1%, ** = 5%, and * = 10%

Table 2 presents the test results for external sources. Quarterly FDI inflows and exports have dropped significantly for Bangladesh, as mean differences are negative and statistically significant. On the other hand, foreign loans have increased significantly during the COVID period, which may indicate the government’s efforts to source more financial resources to support immediate and short to medium-term COVID-19 recovery management. With the increases in foreign loans, quarterly external debt repayments have also increased significantly during the COVID period. A good sign, however, that makes the task easier for Bangladesh is noticeably larger remittance inflows over the COVID period. The country’s foreign reserve has crossed the 43.4 billion USD mark during the period (as of March 2021), due to the overwhelming remittance inflows (Bangladesh Bank 2021). While total ODA receipts appear to rise from pre- to post-COVID, it remains statistically non-significant. On the other hand, Sri Lanka appear to show a statistically significant fall in FDI inflows and exports receipts similar to Bangladesh. Alongside, average quarterly total outstanding debt as % of GDP has increased significantly during the COVID period, which could be the result of lower repayments or increased foreign loans. The exact reason cannot be explored due to the unavailability of data. Sri Lanka’s recent crisis of foreign reserves lower the country’s ability to make debt service payments and repayment of foreign borrowings. Table 2 shows that both Bangladesh and Sri Lanka face a significant fall in financing from external sources through the private sector with an increasing foreign debt, which suggests increasing pressure on the fiscal balance in both countries.

## Are Financing Priorities for SDGs Shifting?

The global economic disturbance caused by the COVID-19 pandemic has substantially affected the world’s commitment and ability to achieve SDGs by 2030 (Shulla et al. 2021). The world’s priorities to achieve SDGs are now placed on the back burner and the need to recover from the economic crisis appears as the first priority in most developing countries (Barbier and Bugges 2020). The increase in poverty, forcing students to undergo online education, implementation of work from home, long periods of home quarantine and high level of stress due to health effects can challenge the achievement of SDGs (Shulla et al. 2021). The COVID-19 pandemic has both explicit and implicit effects on SDGs. While SDG 1. No Poverty; SDG 2. Zero Hunger; SDG 3. Good Health and Well-being; SDG 4. Quality Education; SDG 5. Gender Equality, SDG 8. Decent Work and Economic Growth and SDG 10. Reduced Inequality are affected explicitly, SDG 6. Clean Water and Sanitation; SDG 7. Affordable and Clean Energy; SDG 9. Industry, Innovation and Infrastructure; SDG 11. Sustainable Cities and Communities; SDG 13. Climate Action; SDG 14. Life Below Water; SDG 15. Life on Land; SDG 16. Peace and Justice Strong Institutions are affected implicitly (Afzal 2020).

**Fig. 7:**
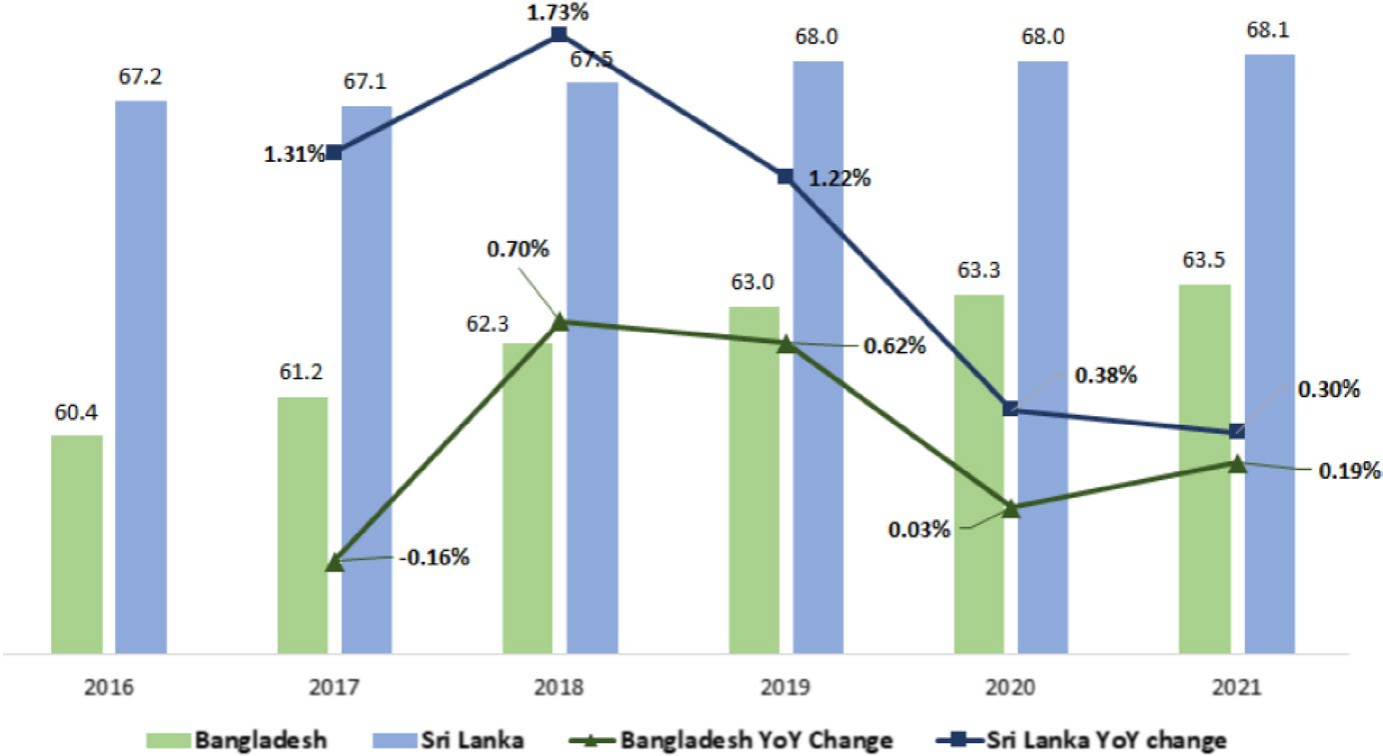
SDG Index Score, 2016–2021. *Source*: Sustainable Development Report, SDG Dashboard (available from https://dashboards.sdgindex.org/profiles/)

Bangladesh and Sri Lanka are among the countries committed to achieve SDGs by 2030. However, the high vulnerability of these two countries to the COVID-19 pandemic and the decline in international support during the last year slowed down the process of achieving SDGs. Moreover, the ability of developing countries to discover innovative and cost-effective methods for policy measures in the near future is uncertain (Barbier and Bugges 2020). Figure 7 shows a declining trend of overall SDG progress of the two countries even from the pre-pandemic year of 2018, after the goals were launched in January 2016. For Sri Lanka particularly, 2020 and 2021 have seen a consistent decline by 0.4% and 0.3%, respectively, while Bangladesh has seen sharp decline by 0.03% in 2020, followed by a slight recovery in 2021. The overall pattern for both countries suggest a significant slowdown in SDG progress.

**Fig. 8:**
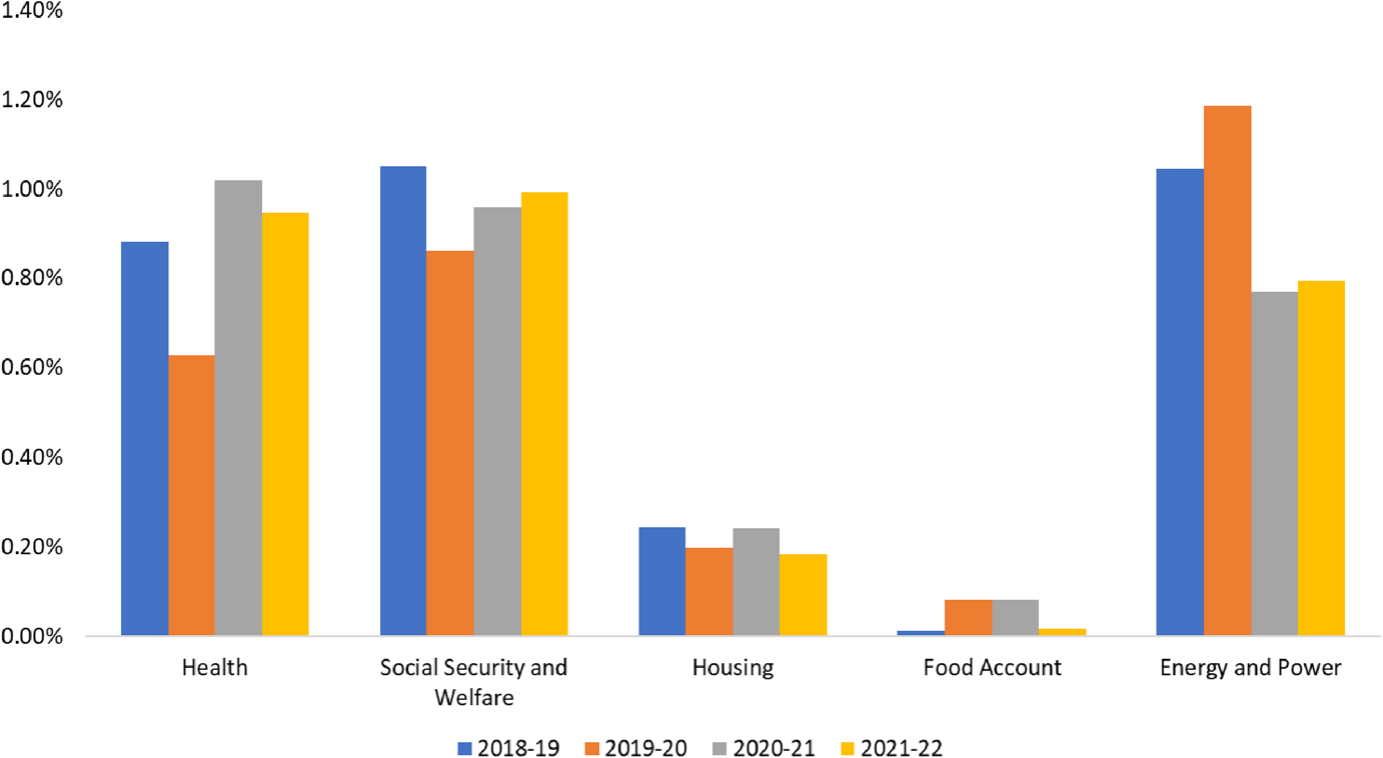
Bangladesh’s key sectoral expenditures as % of GDP. *Source*: Authors’ developed based on Ministry of Finance, GoB data

**Fig. 9:**
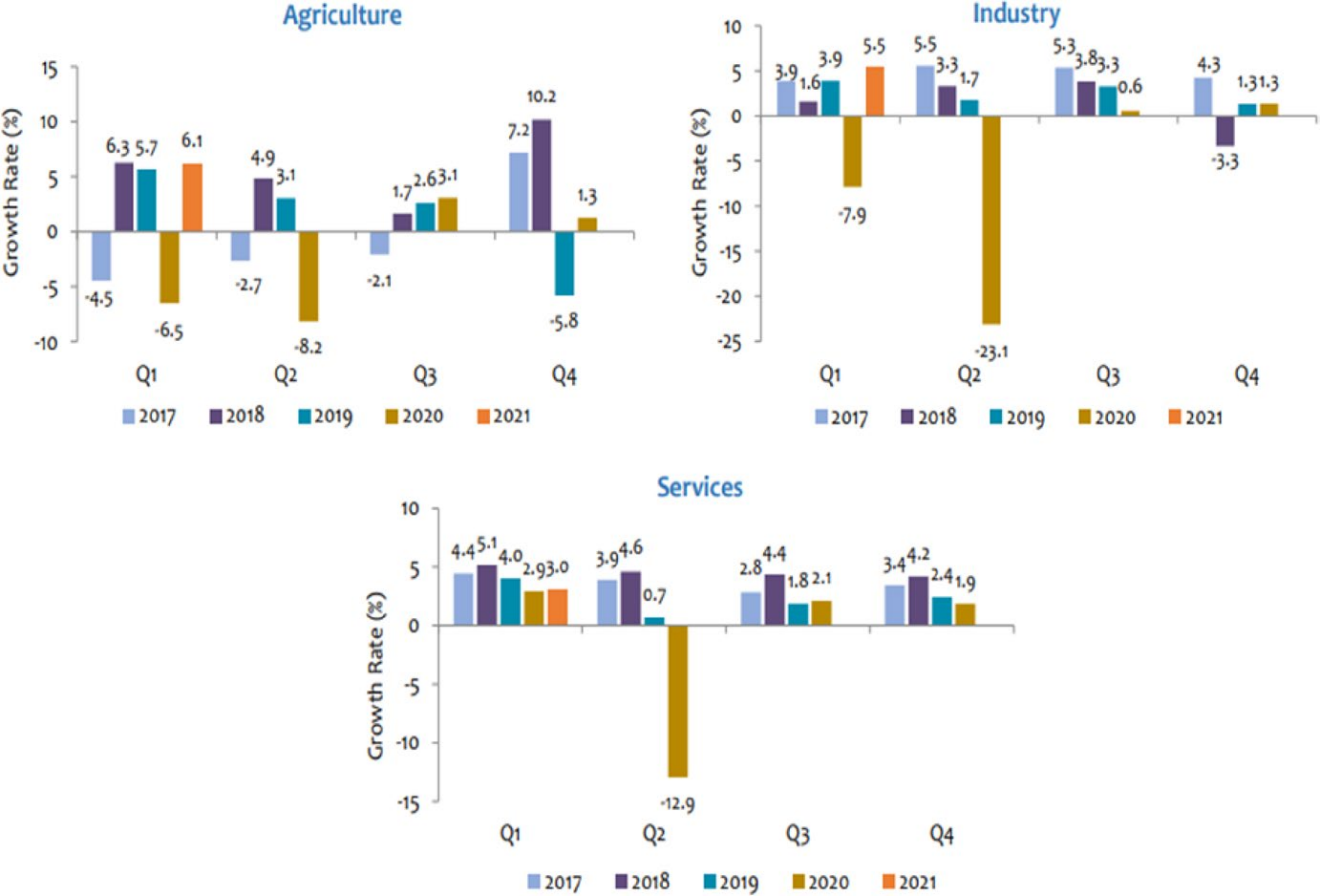
Changes in Sri Lanka’s key sectoral expenditures. *Source*: Department of Census and Statistics, 2021

While public finances from domestic and external sources become tough, the governments become over-reliant on domestic borrowing. During the COVID-19 pandemic, the governments of Bangladesh and Sri Lanka faced a declining trend of external financing and domestic revenues, which forced them to borrow heavily from domestic banking or non-banking sources. With an expanding fiscal deficit and increased domestic borrowing, the governments prioritized saving lives, livelihood and economic recovery by supporting businesses and industries. Both governments have boosted up immediate spending to manage the shock to the health system and to minimize the spread of the diseases, while supporting individuals and businesses directly or indirectly. As we pass the one and half year point of COVID-19 with no sign of returning to a pre-COVID state, the governments in both countries reshuffled their fiscal priority for the coming years. In addition, the priorities remain the same—saving lives as much as possible by improving the health systems and vaccinating citizens, while supporting businesses and industries with stimulus and bailout packages to continue economic momentum. As further financial resources are being diverted to these purposes, priorities assigned for the SDGs during the pre-COVID period appear to gradually fade away. As such, the normal courses of actions and programs to achieve the SDGs by 2030 are not the top most priorities now as they were before the pandemic. Figure 8 shows that health and social welfare expenditures as % of GDP in Bangladesh have increased in the last two years significantly more than other sectors that are closely related to the SDGs, such as energy and power, housing and food account. Furthermore, a wide range of policy responses to the COVID situation also evidenced the topmost priority of the Bangladesh government for saving lives and preventing the economy from going into a recession. In Appendix A1, a summary of the policy responses as of March 2021 is provided (Figs. 8, 9).

Although the government of Bangladesh has taken steps to achieve SDGs through its five-year plan, the implementation of this plan is now at a risk. The high dependency of the country on international donors, NGOs, and banks in achieving its economic and sustainable goals has now led to slow implementation, primarily due to these parties having fewer support during the pandemic. Sakamoto et al. (2020) investigated how the negatives of COVID-19 pandemic could affect SDG priorities in Bangladesh. They mainly analyzed four vulnerable areas; the garment industry, urban slums, social exclusion, and pre-existing health conditions. They identified that these areas are most affected, and a considerable time period will be needed to recover them to the pre-pandemic state. Furthermore, most slum dwellers continued to work in streets during lockdown periods to earn their daily basic needs. The panic buying of high- and middle-income earners also negatively affect low-income earners. These reports highlight the lack of reliefs from the government and NGOs to the low-income population (Sakamoto et al. 2020). Thus, the philosophy of the SDGs; No one left behind, is questionable.

Bangladesh was moderately performing being on track in the first of the 17 goals of SDGs. United Nations (2020) revealed that COVID-19 has started affecting the progress of SDG 2 (Zero hunger) projecting an extreme food deficit for 270 million people. There were also reports that a total of 14 goals is severely hampered by COVID-19 with slightly positive outcomes in Bangladesh. Sunny et al. (2021) explained that the fast-growing fisheries industry in Bangladesh is highly impacted by the pandemic. The closure of infrastructure services, labor calamity, high inflation, sudden health issues and decreased income as a result of the COVID-19 pandemic challenged the progress of SDGs in Bangladesh (Sunny et al. 2021). They pointed out that the SDGs like SDG 1: eliminate poverty, SDG 2: erase hungry, SDG 3: good health and well-being and SDG 12: responsible consumption and productions will be challenging to attain due to the significant reduction in people’s income, nutrition, and food security.

Bangladesh projected that it would cost US$ 928.48 billion for the period 2017–2030 to achieve the targets of SDGs where finance and resource mobilization are considered the key components in the coming years (GED 2018). Experts have also projected the annual cost of achieving the SDGs- US$ 66.32 billion in the same report (Equity 2018). Nevertheless, analyzing the expenditures by the government of Bangladesh as a stimulus package (USD 12.11 billion spent so far) to rejuvenate the various economic sectors of the country is likely to make it challenging for the government to spend on development programs to accelerate the growth of SDG targets. To mitigate the challenge, the government has undertaken a plan where public, private, and external sources are likely to make sectoral contribution of 34%, 41%, and 15% in total financing, respectively, from 2020 to 2030 across different sectors of the SDGs (GED 2020). Unfortunately, the private sector investment is also found less effective and stands around 25% of GDP in the last couple of years, which should be at least 35% per annum (EquityBD 2018).

Similar to Bangladesh, Sri Lanka has prioritized to reroute the distribution of funds to immediate needs of the pandemic, away from tasks aims to achieving SDGs. Ariyapperuma and Abeysekera (2020) emphasized that the SDGs such as no poverty, zero hunger, good health and well-being, quality education, gender equality, decent work and economic growth, reduced inequalities, and peace, justice and strong institutions are the mostly affected SDGs by COVID-19. The key economic sectors such as agriculture, tourism and garment industry are highly exposed and impacted by the preventive mechanisms taken for COVID-19. For a country which is highly depending on tourism and foreign workers, the world crisis has significantly burdened the economy. Figure 9 shows that across the three broad sectors of the Sri Lankan economy, the last quarters of 2020 saw a large decline in public expenditures, reflecting the financing priorities moving toward saving the economy from the pandemic’s adverse impacts.

According to UNDP in Sri Lanka (2020) both economic and social SDGs could be affected by COVID-19, mainly due to the key economic drivers of trade, investments and reduced business activity, tourism and remittances. Furthermore, the elderly, people with disabilities, and low-income workers, who are already vulnerable due to various disparities in the society could be further detrimentally impacted by the pandemic.

The Sri Lankan government had to mobilize a significant amount of additional financing to facilitate online education during pandemic in order to achieve quality education specified in SDG4. As a solution to the closure of schools and higher education institutions, online learning was implemented across the country. However, only 50% of the Sri Lankan population have access to 3G/4G internet connection. There are also rural areas without any network coverages. Therefore, despite the transition to digital delivery, a number of students in rural areas did not have the commodities to accommodate this type of learning.

The recent currency swap agreement for USD200 million with the Bangladesh Bank highlighted the worse economic condition in Sri Lanka. On June 28, 2021, Daily Mirror, a newspaper in Sri Lanka reported that the Central Bank of Sri Lanka printed LKR 23 billion fresh money to inject to the economy. The government of Sri Lanka has taken this step to overcome the liquidity issues in the market as a result of the unexpected island wide lockdown during April to May 2021. However, the newspaper further highlighted that the excessive money print by a developing country like Sri Lanka will cause a high inflation and deficit in the balance of payment (Daily Mirror 2021). Moreover, during a period where citizens in the country are struggling to survive, the increase of fuel price on 11 July (Newsfirst 2021) has also negatively affected the social life of citizens as they navigate out of restrictions. Social actors, media and opposition parties continuously pressurize the government to take immediate remedial actions to overcome this high economic and social disaster.

As a result of these vulnerabilities, the SDGs priorities such as SDG 1 no poverty, SDG 2 zero hunger, SDG 4 quality education, SDG 5 gender equality and SDG 8 decent work and economic growth can be explicitly and negatively affected, while all the other SDGs can affect implicitly. However, it should be acknowledged that the COVID-19 pandemic has forced the two countries to significantly increase spending for public health and investments in the health systems development, which the governments never considered as a priority in the pre-pandemic era. Over a longer period, the significant expansion of healthcare and investments in health quality improvement in the advent of the pandemic could help the countries positively in achieving the SDG 3 Good Health and Well-being.

## Conclusion

The COVID-19 pandemic is threatening economies across the world irrespective of the development status of the countries. The pandemic pressurizes the society as a threat on their lives as well as an economic burden. With the closure of borders by the country for air and marine travel, the concept of globalization has been highly challenged and the open foreign trade market badly hit.

Prior to 2020, world leaders and officials prioritized the rising concern about climate change. To combat this among other inequalities around the world, they agreed-up on 17 SDGs to achieve by 2030. Both developed and developing countries identified achieving these SDGs as a top priority and published them as a separate policy. However, the emergence of the COVID-19 pandemic brought a paradigm shift in the governments' policy priorities, particularly in developing countries. This is because the pandemic’s harshest impacts are faced by developing economies. We investigated the evolution of COVID-19, its impact on public financing and how public funding priorities have shifted as a result of the pandemic using a comparative study for two developing countries in South Asian context: Bangladesh and Sri Lanka.

Using content analysis and descriptive statistics, we obtained mixed results for two countries. Overseas Development Assistants have declined Q1 2020 through Q1 2021 compared to the previous years in both countries. Further, FDI inflows and exports receipts also declined in the middle of 2020, with signs of repossession in the following quarters. However, workers remittances and total tax revenues of Bangladesh has continuously improved in contrast to the case of Sri Lanka. The continuous decline of main government revenue sources in Sri Lanka causes fiscal struggles for a prolonged period.

Overall, both Bangladesh and Sri Lanka face a significant fall in financing from external sources through private sector with an increasing foreign debt, which suggests increasing pressure on the fiscal balance in both countries. Moreover, our analyses demonstrate that the SDG are now being deprived due to low-income flows and shifting available funds from SDG to immediate needs of the country. Both economic and social SDGs are affected by the pandemic and the philosophy of SDGs; No one left behind, is now disputed.

There are several policy implications from this study. First, developing countries should consider methods for attracting international reliefs, especially in terms of donations, to lessen poverty and hunger. Additionally, proper procedures should be implemented to vaccinate a majority of people in the country using foreign donations. Second, the fiscal policy should be sound and stable for a considerable period, without giving a high burden to the general public. Unnecessary tax concessions can uplift while reducing the financing flows to less prioritized projects. Third, vulnerable economies can negotiate for short-term loan moratoriums with foreign loan providers until the country economically recovers. Finally, a proper policy should be implemented which facilitate achieving SDGs without compromising them for short-term vulnerabilities.

However, the unavailability of post-COVID data limits the scope of this paper. Future research could analyze the impact of the COVID-19 pandemic on SDG priorities with econometric analysis once this data becomes available. While restoring health becoming an utmost priority, regardless of the state of the development, governments should take policy solutions to prevent massive economic shocks that can be caused by paralyzed economic activities during multiple lockdowns and restrictions. Particularly developing economies are facing pressing challenges of restoring critical supply chains especially for foods and medicine, financing businesses through various stimulus packages, curbing unemployment levels, enhancing the livelihood of people by assuring stable income for the workforce and their families and regaining income from foreign trade and recovering the revenue loss of the tourism industry. Since most of the developing nations are suffering from external debt burden, the governments should discuss with the lending countries and institutions to restructure debt payments in an event country is unable to meet liquidity needs for debt servicing. To have a better economic prospect, policymakers should revisit SDG and prioritize them to allocate resources efficiently for transformation beyond COVID-19. Government should encourage financial intermediaries who play a vital role particularly in regional areas to inject more liquidity to different community groups which in turn improve consumption and facilitate businesses to reopen. Tax incentives and waiving penalties for private sector will stimulate the economy by bringing business back to their pre-pandemic levels.

## Supplementary Information

Below is the link to the electronic supplementary material.

Supplementary file1 (DOCX 15 KB)
