# Supplementary material for: COVID-19 Effects on Public Finance and SDG Priorities in Developing Countries: Comparative Evidence from Bangladesh and Sri Lanka
Source: Eur J Dev Res. 2022 Jul 27;35(1):85–111. doi: 10.1057/s41287-022-00558-6 (PMC9330931; doi:10.1057/s41287-022-00558-6)
Supplement: Supplementary file 1 — Supplementary file1 (DOCX 15 KB) [file 41287_2022_558_MOESM1_ESM.docx]

**Appendix 1: Bangladesh policy responses to COVID-19 pandemic**

| **Types of responses** | **Policy responses details** |
| --- | --- |
| **Fiscal** | March 2020  > Tk. 2.5 billion in additional resources to fund COVID-19 Preparedness and Response Plan and expand the existing transfer programs that benefit the poor. Increased allocation was made to the Open Market Sale program to facilitate the purchase of rice at one-third the market price.  > Announced a Tk. 50 billion (about US$ 588 million) stimulus package for exporting industries to be channeled through Bangladesh Bank (BB) and distributed by the commercial banks at a 2 percent service charge.  > Subsidizing interest payments on working capital loans of up to Tk. 600 billion (about US$ 7.1 billion) provided by scheduled banks to businesses.  April 2020  > Announced the allocation of Tk. 21.3 billion (about US$ 250.9 million) under a housing scheme for the homeless, Tk.15 billion (about US$ 176.7 million) for the poor who faced job losses from the pandemic, Tk.7.5 billion (about US$ 88.3 million) to provide health insurance for government employees most at risk, and Tk. 1 billion (about US$ 11.8 million) in bonus payments for public health workers treating COVID-19 patients.  > Announced Tk. 20 billion (about US$ 235.6 million) in interest payments on behalf of 13.8 million loan recipients negatively impacted by the national shutdown will be covered by the government.  January 2021  > Increased the COVID-19 Emergency Response and Pandemic Preparedness Project costs by Tk 56.6 billion (about US$ 666.7 million) mostly reflecting the procurement, preservation, and distribution of vaccines.  > Announced two additional stimulus packages - Tk. 15 billion for the micro credit and marginal people's lifestyle development program, and Tk. 12 billion for the old age and widow allowance expansion program.  May 2021  > the government announced its second-round cash assistance program of Tk. 9.3 billion for the targeted population who lost their jobs from the ongoing lockdown. Thus far, Tk. 390.7 billion (about US$ 4.6 billion) of fiscal stimulus has been announced, of which Tk.186 billion (about US$ 2.2 billion) has been disbursed as of end-April, 2021.  > The National Board of Revenue has suspended duties and taxes on imports of medical supplies, including protective equipment and test kits.  June 2021  > The FY22 Budget includes higher allocations (in Taka) for health, agriculture, and social safety net programs, although effective targeting remains a challenge.  > As a precautionary measure, the government has decided that 25 percent of budgetary allocations for development projects will be placed on hold, affecting low-priority projects. It has approached donors seeking budget support. |

**Appendix 1 (Continued): Bangladesh policy responses to COVID-19 pandemic**

| **Types of responses** | **Policy responses details** |
| --- | --- |
| **Monetary and Macro-financial** | > BB announced the purchase of treasury bonds and bills from banks.  > The repo rate was successively cut from 6 percent to 4.75 percent over three cuts from March to July.  > The cash reserve ratio (CRR) for banks was reduced on both a daily (from 5 to 3.5 percent) and a bi-weekly basis (from 5.5 to 4 percent).  > The CRR was also cut for offshore banking operations, effective July 1, and for Non-Bank Financial Institutions (NBFIs), effective June 1.  > The advance-deposit ratio and investment-deposit ratio was raised by 2 percent to facilitate credit to the private sector and improve liquidity.  > The Export Development Fund was raised from US$ 3.5 billion to US$ 5 billion, with the interest rate slashed to 1.75 percent and the refinancing limit increased.  > BB has created several refinancing schemes totaling Tk 415 billion (about US$ 4.9 billion), a 360-day tenor special repo facility, and a credit guarantee scheme for exporters, farmers, and SMEs to facilitate the implementation of the government's stimulus packages.  > In addition, BB has taken measures to delay non-performing loan classification, relax loan rescheduling policies, waive credit card fees and interests, suspend loan interest payments, relax credit risk rating rules for banks, extend tenures of trade instruments, lower farm loan interest rate and allow short-term farm loan rescheduling, and ensure access to financial services.  > BB imposed an additional 1.0 percent general provision against loans that have enjoyed deferral/time extension facilities. |
| **Exchange Rate and Balance of Payment** | > Foreign exchange rules were eased by BB to: (i) provide foreign currency to Bangladeshi nationals facing problems while returning home due to travel disruptions; and (ii) allow foreign owned/controlled companies operating in Bangladesh to access short term working capital loans from their parent companies/shareholders abroad to meet actual needs for payments of wages and salaries.  > International factoring was introduced to accelerate exports. BB has been intervening in the foreign exchange market to keep the exchange rate relatively stable following the COVID-19 outbreak. |

Source: IMF Policy Tracker Database (as of 28 October 2021)
